# [^177^Lu]Lu-AKIR001 for CD44v6-Positive Pancreatic Cancer: Preclinical Efficacy and Combination Strategies

**DOI:** 10.2967/jnumed.125.271705

**Published:** 2026-09

**Authors:** Amanda Gustafsson, Hedvig Svedberg, Sara S. Rinne, Filippa Bertilsson, Ram Kumar Selvaraju, Anja C. Lundgren Mortensen, Cecilia Lindskog, Marika Nestor

**Affiliations:** 1 Department of Immunology, Genetics and Pathology, Uppsala University, Uppsala, Sweden;; 2 Science for Life Laboratory, Uppsala University, Uppsala, Sweden;; 3 Department of Medicinal Chemistry, Uppsala University, Uppsala, Sweden; and; 4 Department of Molecular Medicine and Surgery, Karolinska Institutet, Stockholm, Sweden

**Keywords:** radiopharmaceutical therapy, pancreatic ductal adenocarcinoma, PDAC, radiosensitization, CD44v6

## Abstract

Pancreatic ductal adenocarcinoma (PDAC) is highly aggressive, with a 5-y survival rate of less than 5% for patients with advanced disease. CD44v6 is frequently overexpressed in the malignancy, representing a promising therapeutic target. Here, we evaluated the efficacy of [^177^Lu]Lu-AKIR001, a CD44v6-targeting radiopharmaceutical, alone and combined with chemotherapy for the treatment of PDAC. **Methods:** CD44v6 expression, radioligand binding, and chemotherapy sensitivity were assessed in PDAC cell lines. Mice bearing PDAC xenografts received [^177^Lu]Lu-AKIR001, chemotherapy, or a combination of the two modalities. Toxicity was determined by body weight monitoring and hematologic and organ analyses. **Results:** Three of the 4 cell lines expressed CD44v6. In vivo, tumor uptake exceeded 100 %IA/g. Tumor growth inhibition was activity-dependent, with complete remissions detected after the administration of 12 MBq of [^177^Lu]Lu-AKIR001 (40%) and 4 MBq of [^177^Lu]Lu-AKIR001 combined with paclitaxel (14%). **Conclusion:** [^177^Lu]Lu-AKIR001 demonstrated activity-dependent therapeutic potential in mice bearing PDAC xenografts. Combination therapy with paclitaxel indicated potential synergistic benefit, but further investigation and optimization are warranted.

## Visual Abstract

**Figure:**
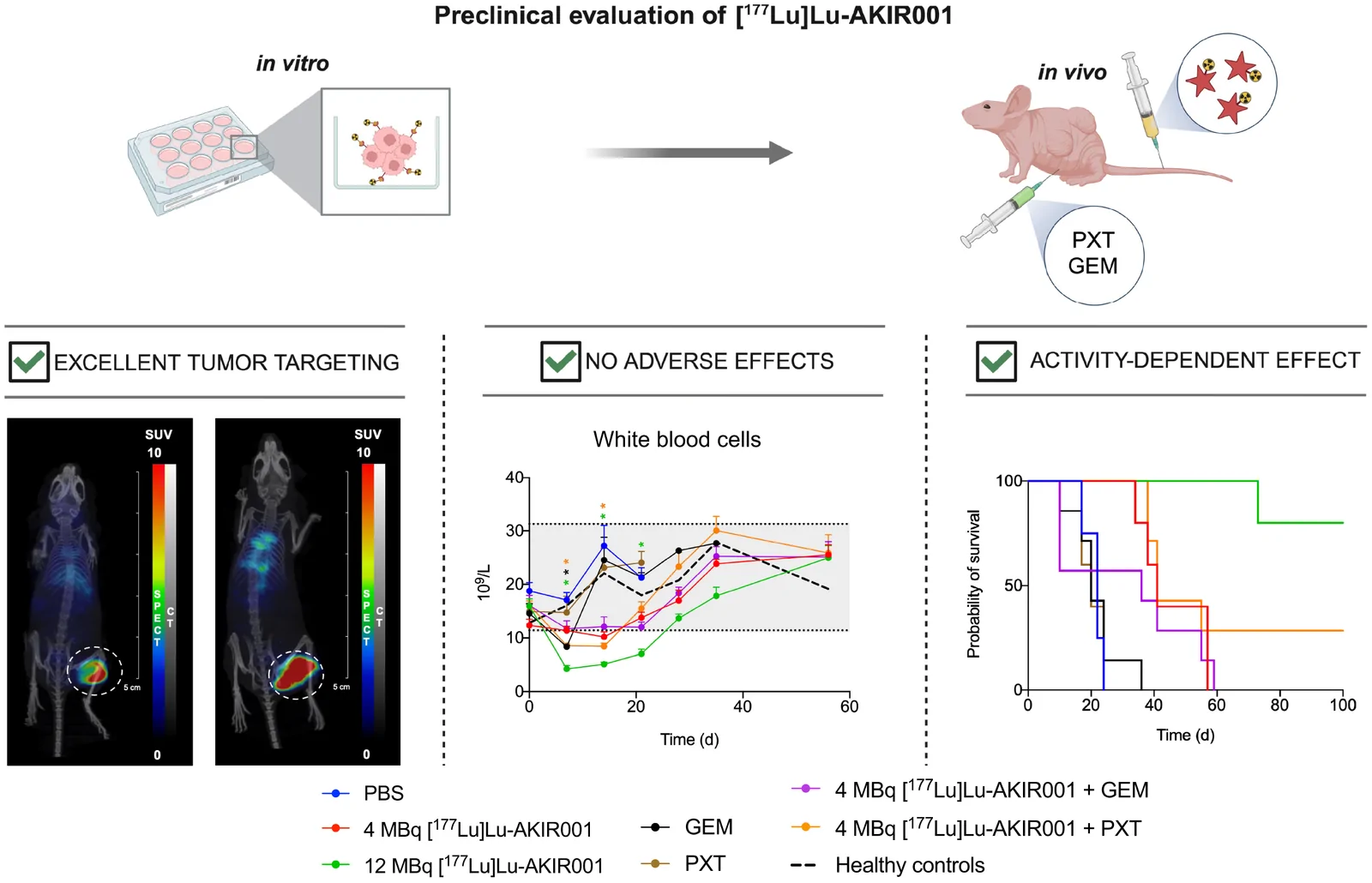

Pancreatic ductal adenocarcinoma (PDAC) accounts for over 90% of pancreatic cancer cases and remains one of the most lethal malignancies, with a 5-y survival rate of less than 5% in patients with metastatic disease (1,2). Although surgical resection is the only curative approach, it is feasible only in 10%–20% of patients with local disease (1,3). To date, systemic chemotherapy remains the mainstay of treatment for advanced PDAC, prolonging survival by no more than 6 mo for single-agent therapies or up to a year with multiagent combinations (4,5).

Paclitaxel and gemcitabine are widely used chemotherapeutics in PDAC. Gemcitabine disturbs DNA synthesis by incorporation into DNA and reduction of intracellular deoxynucleotide triphosphate pools (6). Paclitaxel induces mitotic arrest by disrupting microtubule function (7).

Radiopharmaceutical therapy (RPT) enables selective delivery of cytotoxic radiation to tumors and may overcome the limitations of external beam radiotherapy in metastatic or anatomically challenging disease (8). Recently, CD44v6-targeted RPT using [^177^Lu]Lu-AKIR001 demonstrated therapeutic efficacy in various xenograft models (9). [^177^Lu]Lu-AKIR001 is currently under clinical investigation for the treatment of several malignancies (NCT06639191).

Given the substantial unmet clinical need in PDAC, we evaluated the therapeutic efficacy of [^177^Lu]Lu-AKIR001 alone and in combination with gemcitabine or paclitaxel.

## MATERIALS AND METHODS

All methods are described in detail in the supplemental materials, available at http://jnm.snmjournals.org. All materials used are listed in Supplemental Table 1. AKIR001 was conjugated with p-SCN-Bn-DOTA (GenScript ProBio) and radiolabeled with ^177^Lu. Four human pancreatic cancer cell lines were evaluated in vitro. The biodistribution and therapeutic effect of [^177^Lu]Lu-AKIR001 were studied in BALB/c nu/nu mice bearing BxPC3 xenografts. Potential toxicity was evaluated by hematologic and organ analyses and body weight monitoring (10).

## RESULTS

### Stability of [^177^Lu]Lu-AKIR001

Stability of [^177^Lu]Lu-AKIR001 was evaluated by instant thin-layer chromatography in phosphate-buffered saline, mouse serum, and in the presence of a 500-fold molar excess of ethylenediaminetetraacetic acid. The radioconjugate was found to be stable (>95% intact radioconjugate) for at least 48 h (Table 1).

**TABLE 1.** Stability of [^177^Lu]Lu-AKIR001 Over Time

	% Stability		
Time (h)	PBS	Serum	EDTA
1	102.3 ± 1.8	100.6 ± 2.2	101 ± 1.9
24	100.0 ± 4.8	98.2 ± 4.9	99.0 ± 1.8
48	97.5 ± 3.9	97.0 ± 1.0	95.4 ± 0.9

PBS = phosphate-buffered saline; EDTA = ethylenediaminetetraacetic acid.

Data expressed as mean *±* SD.

### In Vitro Cell Line Characterization

**FIGURE 1.**
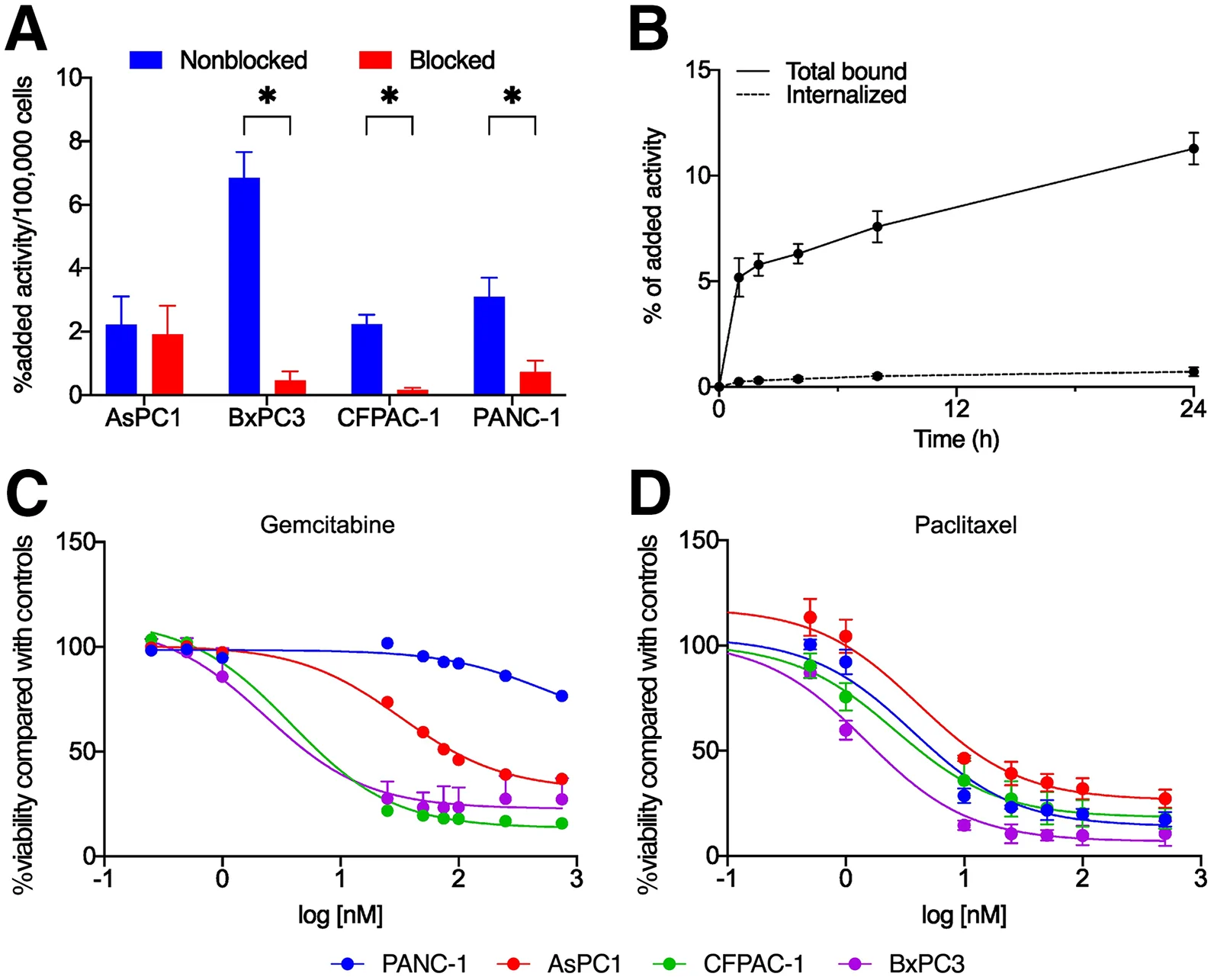
In vitro characterization. (A) [^177^Lu]Lu-AKIR001 binds specifically to PANC-1 (*N* = 3, *n* = 4), CFPAC-1 (*N* = 3, *n* = 4), and BxPC3 (*N* = 3, *n* = 4) but not to AsPC-1 (*N* = 3, *n* = 4). (B) Internalization of [^177^Lu]Lu-AKIR001 in BxPC3 cells (*N* = 3, *n* = 3). Cell viability after treatment with 0–750 nM of gemcitabine (*N* = 3, *n* = 6) (C) or 0–500 nM of paclitaxel (*N* = 3, *n* = 6) (D). Error bars represent SD. Some error bars are smaller than symbols and therefore not visible. *P = 0.05.

Specific binding was observed in 3 of the 4 tested cell lines (Fig. 1A), with a picomolar affinity of the radioligand to its target (Table 2). Internalization in BxPC3 cells was less than 6% of total bound [^177^Lu]Lu-AKIR001 (Fig. 1B), and cells were more sensitive to paclitaxel than to gemcitabine (Fig. 1C).

**TABLE 2.** Association Constant (*k*_a_), Dissociation Constant (*k*_d_), and Binding Affinity (*K*_D_) of [^177^Lu]Lu-AKIR001 in 3 PDAC Cell Lines

Variable	BxPC3	PANC-1	CFPAC-1
*k*_a_ (1/(M*s))	3.9 × 10^5^ ± 4.7 × 10^4^	5.1 × 10^5^ ± 3.3 × 10^5^	1.7 × 10^6^ ± 4.9 × 10^4^
*k*_d_ (1/s)	1.4 × 10^−7^ ± 2.4 × 10^−7^	2.5 × 10^−5^ ± 3.8 × 10^−6^	4.1 × 10^−5^ ± 1.3 × 10^−5^
*K*_D_ (pM)	0.4 ± 0.7	26.7 ± 9.5	24.9 ± 7.6

Data expressed as mean *±* SD.

### Biodistribution of [^177^Lu]Lu-AKIR001

**FIGURE 2.**
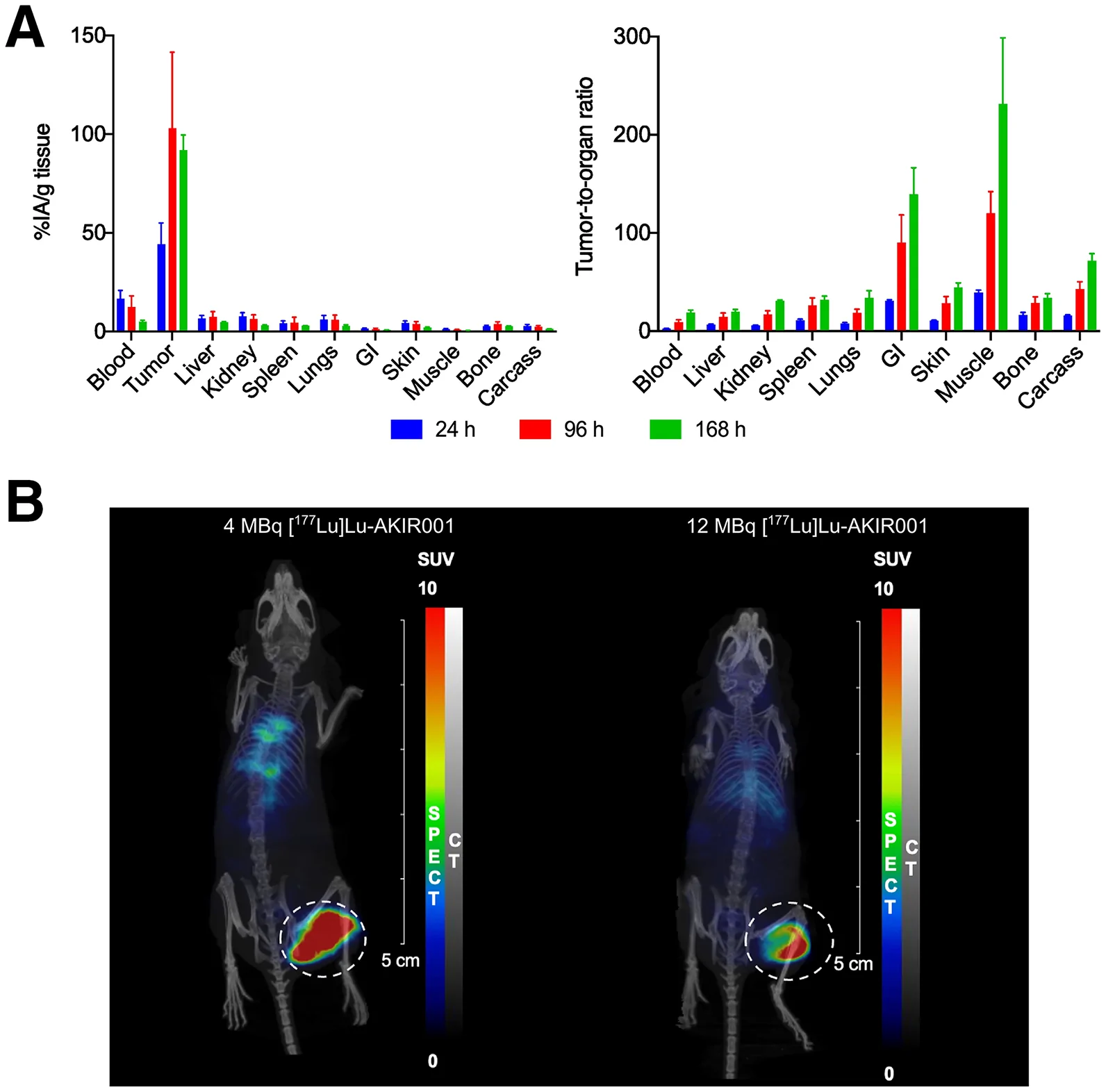
Biodistribution of [^177^Lu]Lu-AKIR001. (A) Mice (*N* = 12) bearing BxPC3 tumors were injected with [^177^Lu]Lu-AKIR001 and dissected at 24 (*N* = 4), 96 (*N* = 4), and 168 h (*N* = 4) postinjection. Error bars represent SD. (B) Representative SPECT/CT images at 96 h postinjection. GI = gastrointestinal.

Ex vivo biodistribution of [^177^Lu]Lu-AKIR001 in BxPC3 xenografts demonstrated peak tumor uptake at 96 h postinjection (Fig. 2A), further visualized by SPECT/CT (Fig. 2B).

### Therapeutic Efficacy of [^177^Lu]Lu-AKIR001

Therapeutic efficacy of [^177^Lu]Lu-AKIR001 alone and in combination with chemotherapy was assessed in BALB/c nu/nu mice bearing BxPC3 tumors (Supplemental Fig. 1). There was no significant difference in tumor volume between groups at treatment initiation (Supplemental Fig. 2).

**FIGURE 3.**
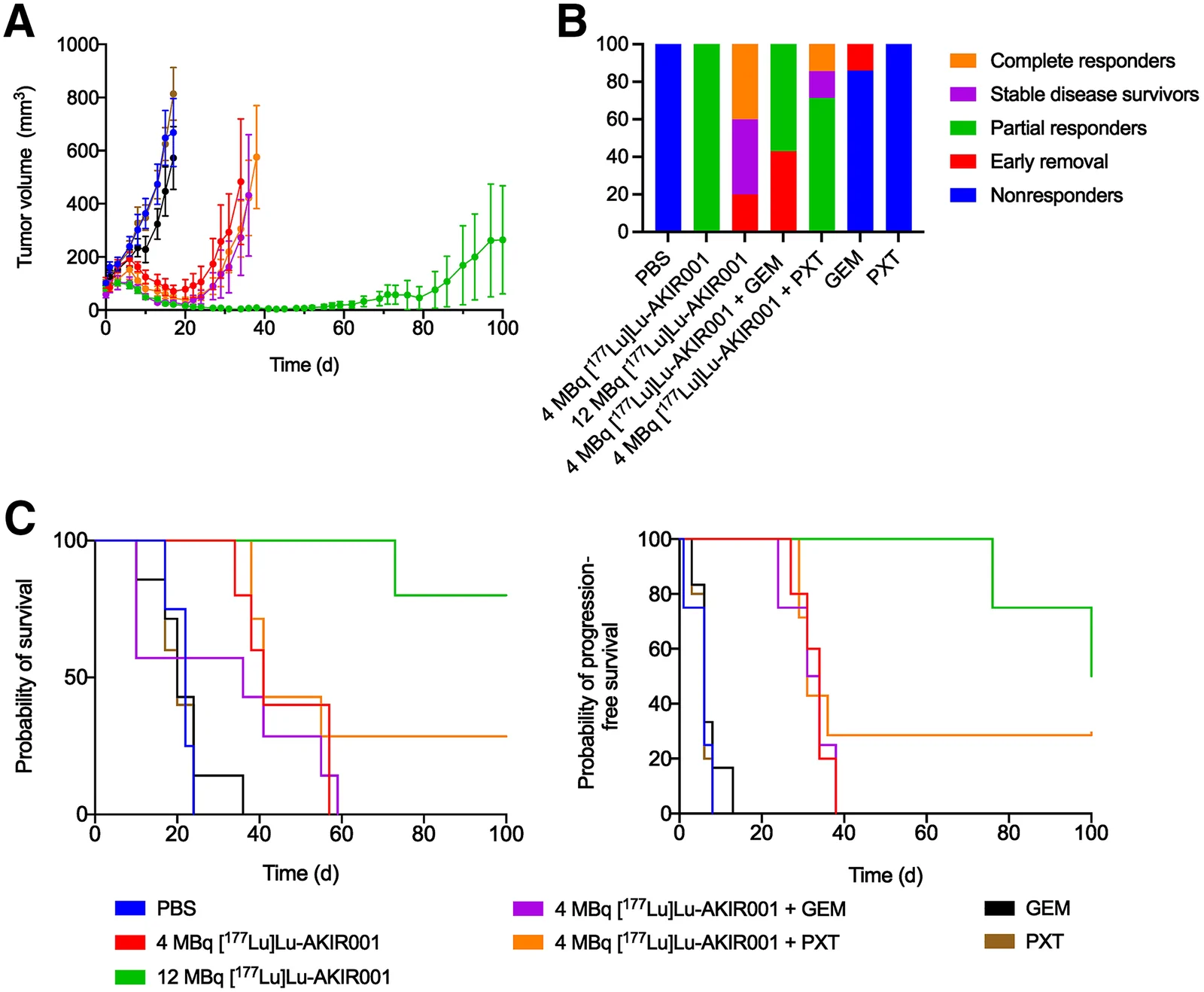
Therapeutic efficacy of [^177^Lu]Lu-AKIR001. (A) Mean ± SEM tumor volumes over time until first animal in each group (*N* ≥ 4 for xenografts) was euthanized (early removal or tumor-bearing limit). (B) Distribution of animals by treatment group and response category: complete responders (complete tumor remission), stable disease survivors (tumors present but alive at day 100), partial responders (tumor growth delayed relative to controls, but tumor-bearing limit reached), early removal (euthanized because of toxicity or ulceration), and nonresponders (no measurable treatment effect). (C) Kaplan-Meier survival analysis (including animals that were removed early) and progression-free survival (relative tumor volume > 2 when compared with treatment initiation) on animals with evaluable tumor outcome. GEM = gemcitabine; PBS = phosphate-buffered saline; PXT = paclitaxel.

Activity-dependent tumor growth inhibition was observed, together with complete remission after the administration of 12 MBq of [^177^Lu]Lu-AKIR001 monotherapy and the combination of 4 MBq of [^177^Lu]Lu-AKIR001 with paclitaxel (Fig. 3; Supplemental Fig. 3). Parameters characterizing treatment efficacy, including median survival and median progression-free survival, are presented in detail in Supplemental Table 2. Immunohistochemistry verified CD44v6 expression in tumor tissue (Supplemental Fig. 4).

### Assessment of Treatment-Related Toxicity

**FIGURE 4.**
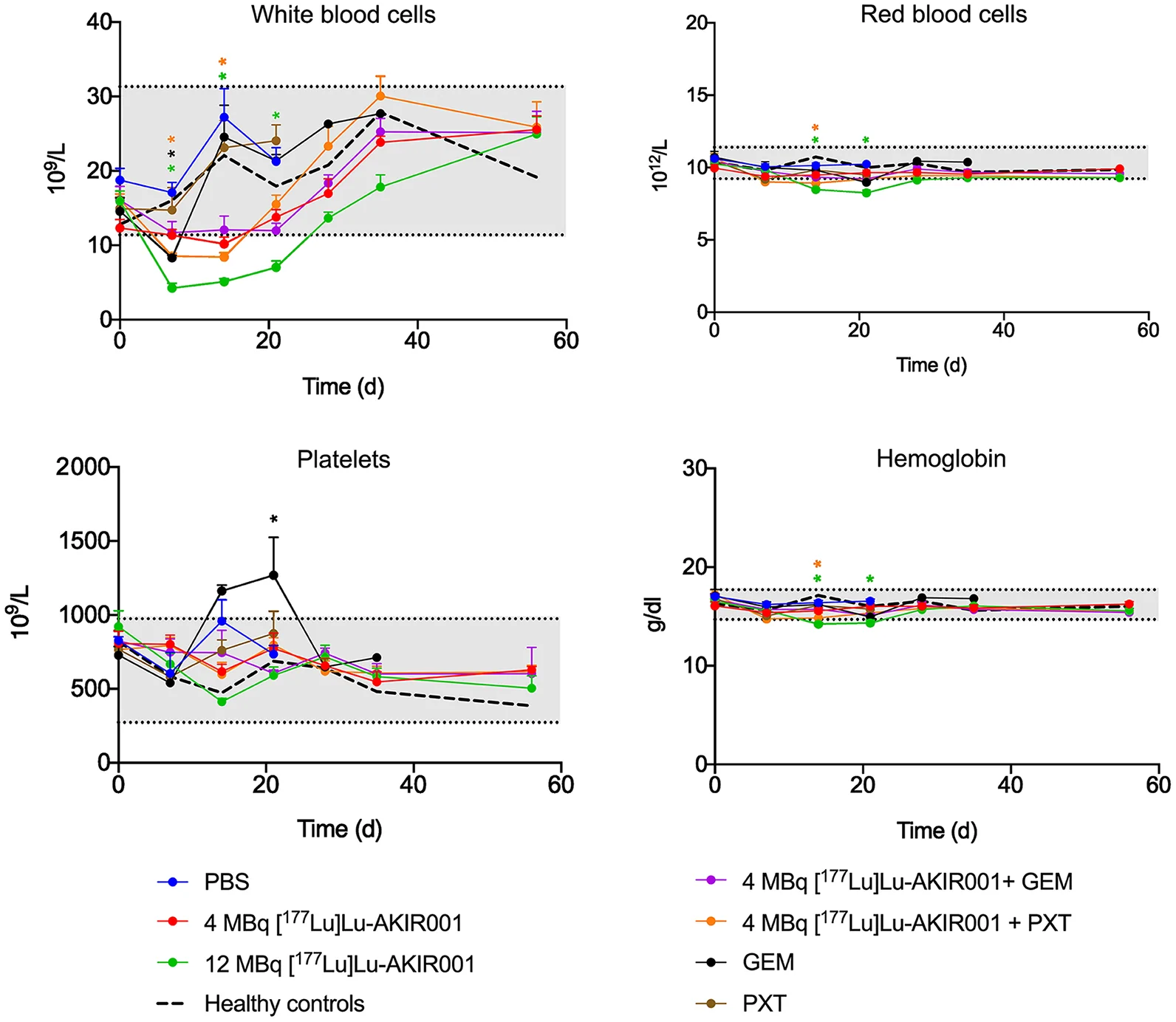
Evaluation of hematologic toxicity (*N* ≥ 4 for xenografts). Monitoring of white blood cells, platelets, hemoglobin, and red blood cells was performed throughout study. Data are presented as group mean ± SEM. *Indicates significant difference detected compared with both phosphate-buffered saline group and littermate controls (*P* < 0.05). GEM = gemcitabine; PBS = phosphate-buffered saline; PXT = paclitaxel.

A transient reduction in red blood cells, white blood cells, and hemoglobin was observed, most notably after 12 MBq of [^177^Lu]Lu-AKIR001 (Fig. 4). No significant differences in organ–to–brain weight or organ–to–body weight ratios were detected when compared with controls (Supplemental Figs. 5A and 5B). Body weight remained stable for all but the gemcitabine-treated mice (Supplemental Fig. 5C).

## DISCUSSION

Building on our preclinical validation of [^177^Lu]Lu-AKIR001 (9), we observed potent antitumor activity, indicating a potential benefit when combined with paclitaxel in a preclinical PDAC model. RPT activities were based on previous work (9), and chemotherapy scheduling aligned peak tumor uptake of [^177^Lu]Lu-AKIR001 with chemotherapy-induced cell cycle arrest (11–14).

The combination of radiopharmaceuticals with chemotherapy has been investigated, including [^177^Lu]Lu-DOTATATE with capecitabine/temozolomide and [^177^Lu]Lu-PSMA-617 with cabazitaxel (15–17). The antibody-based [^177^Lu]Lu-J591 plus docetaxel achieved a decline of greater than 50% in prostate-specific antigen levels in 73% of patients with metastatic castration-resistant prostate cancer (18).

[^177^Lu]Lu-AKIR001 potently suppressed tumor growth in mice with PDAC xenografts, with complete remission after 12 MBq of monotherapy (40%) and 4 MBq of [^177^Lu]Lu-AKIR001 combined with paclitaxel (14%) (Fig. 3). Transient hematologic effects occurred at the highest activity but resolved within a few weeks (Fig. 4). The efficacy differences between combinations may reflect the mechanism of action of the chemotherapeutic agents: paclitaxel increases the fraction of cells in the G2/M radiosensitive cell cycle phase and prolongs DNA damage persistence (7), whereas gemcitabine increases S-phase occupancy, potentially limiting synergy with a continuous low-dose of RPT (19).

AKIR001 lacks cross-reactivity with murine CD44v6, as previously reported (9,20). Gross indicators of off-target toxicity were therefore evaluated by blood sampling, body weight monitoring, and the comparison of organ–to–body weight and organ–to–brain weight ratios for initial assessment of target-independent toxicity (10). Although AKIR001 is expected to clear primarily through the liver (21), biodistribution showed low hepatic uptake (Fig. 2A), consistent with the absence of liver toxicity in organ analyses (Supplemental Fig. 5). Importantly, the bone marrow is the most clinically relevant organ at risk, and its evaluation remains feasible in our model.

A 12-MBq dose of [^177^Lu]Lu-AKIR001 produced strong therapeutic effects alone, but combination therapies remain relevant to mitigate resistance and to determine whether comparable outcomes can be achieved with lower doses of radiation, potentially reducing toxicity. In PDAC, such approaches may be most effective early in the disease course, before chemoresistance develops. While no clear additive or synergistic effects were observed, combination therapy with paclitaxel showed indications of improved efficacy, which may be enhanced by optimizing dosing or sequencing. Notably, prior combination of [^177^Lu]Lu-AKIR001 with cisplatin produced promising results in A431 xenografts (9), supporting continued exploration of chemotherapy as an adjunct to RPT, including potential applications in PDAC.

## CONCLUSION

[^177^Lu]Lu-AKIR001 demonstrated potent inhibition of PDAC tumor growth, and coadministration with paclitaxel suggested a potential synergistic benefit. Overall, these findings support continued preclinical and clinical development of [^177^Lu]Lu-AKIR001 for CD44v6-positive malignancies, including PDAC.

## DISCLOSURE

This study was supported by The Sjöberg Foundation (2024-914, 2023-630), the Swedish Cancer Society (Cancerfonden 24 3485 Pj, 22 2391 S), the Erling-Persson Foundation (2023 0120), and the Swedish Research Council (Vetenskapsrådet, 2024-03447). Anja Lundgren Mortensen and Marika Nestor are cofounders of Akiram Therapeutics AB, which owns the intellectual properties of the AKIR001 antibody. No other potential conflict of interest relevant to this article was reported.

## Notes

KEY POINTS **QUESTION:** Is [^177^Lu]Lu-AKIR001 a potential future treatment option for patients with PDAC? **PERTINENT FINDINGS:** In a preclinical study evaluating [^177^Lu]Lu-AKIR001 as a potential treatment for PDAC, therapeutic efficacy was observed in an activity-dependent manner. Potential synergistic benefits were observed after combination with paclitaxel. **IMPLICATIONS FOR PATIENT CARE:** [^177^Lu]Lu-AKIR001 has the potential to be a clinically significant treatment for patients with difficult-to-treat malignancies, supporting the ongoing clinical investigation of AKIR001 (NCT06639191).
